# Trends in the Management and Outcomes of Kidney Transplantation for Autosomal Dominant Polycystic Kidney Disease

**DOI:** 10.1155/2014/675697

**Published:** 2014-08-03

**Authors:** Madhukar S. Patel, Praveen Kandula, David Wojciechowski, James F. Markmann, Parsia A. Vagefi

**Affiliations:** ^1^Department of Surgery, Massachusetts General Hospital, Harvard Medical School, 55 Fruit Street, White 544b, Boston, MA 02114, USA; ^2^Department of Medicine, Saint Barnabas Medical Center, Livingston, NJ 07039, USA; ^3^Department of Medicine, University of California, San Francisco, San Francisco, CA 94143, USA

## Abstract

*Background.* Autosomal dominant polycystic kidney disease (ADPKD) is the most common genetic disorder leading to end-stage renal failure. The objective of this study was to evaluate a longitudinal experience of kidney transplantation for ADPKD.* Methods.* A single center retrospective review of patients undergoing kidney transplantation was conducted, with comparisons across two time periods: early (02/2000–04/2007, *n* = 66) and late (04/2007–08/2012, *n* = 67).* Results.* Over the 13.5-year study period, 133 patients underwent transplantation for ADPKD. Overall, no significant difference between the early and late group with regard to intraoperative complications, need for reoperation, readmissions within 30 days, delayed graft function, and mortality was noted. There was a trend towards increase in one-year graft survival (early 93.1% versus late 100%, *P* = 0.05). In the early group, 67% of recipients had undergone aneurysm screening, compared to 91% of recipients in the late group (*P* < 0.001).* Conclusions.* This study demonstrates consistent clinical care with a trend towards improved rates of one-year graft survival. Interestingly, we also note a significantly higher use of cerebral imaging over time, with the majority that were detected requiring surgical intervention which may justify the current practice of nonselective radiological screening until improved screening criteria are developed.

## 1. Introduction

Autosomal dominant polycystic kidney disease (ADPKD) is a disorder characterized by the development of renal cysts that may result in end-stage renal failure. Two main genes,* PKD1 *and* PKD2*, are thought to be involved in the majority of cases [1]. Variability in the genetic phenotype of ADPKD patients, however, is thought to be due to the range of different genetic mechanisms as well as environmental factors thought to play a role in phenotypic expression [1]. In addition to affecting the kidneys, ADPKD has a number of extrarenal manifestations including cystic formation in other organs such as the liver, seminal vesicles, pancreas, and arachnoid membrane; vascular malformations such as intracranial aneurysms, thoracic aorta dissections, and coronary artery aneurysms; cardiac manifestations such as mitral valve prolapse; and a higher incidence of colonic diverticulosis and diverticulitis [1].

In those with end-stage renal disease, it is accepted that transplantation is the preferred treatment for ADPKD [1]. Of the patients on the kidney transplant waiting list as of December 31 2011, 7256 (8.4%) were listed due to cystic kidney disease and of the 16,055 renal transplants performed in 2011, 2057 (12.8%) were done for patients with cystic kidney disease, with 1,189 from deceased donors and 868 from living donors [2]. In patients undergoing transplantation for ADPKD, current reports in the literature debate the timing and need for native nephrectomy [3–14] as well as the operative approach for native nephrectomy [14–17]. Additionally, due to the potential for catastrophic outcomes perioperatively of undiagnosed cerebral aneurysms, screening for their presence has been suggested at least among subgroups of ADPKD patients [18]. However, there is no clear consensus on management regarding need, timing, or approach to nephrectomy as well as for screening for cerebral aneurysms before undergoing kidney transplantation but rather decisions appear to be patient specific and vary as a function of time and center/surgeon preference. Given this, the objective of this report was to study the longitudinal experience of ADPKD and kidney transplantation at a single center in order to (1) define overall outcomes with management of this disease and (2) identify changes in practice and outcomes over time.

## 2. Methods

A single center retrospective review of consecutive ADPKD patients undergoing transplants from 02/2000 to 08/2012 was conducted. Data collected included recipient demographics, donor type, timing of native nephrectomy if performed (prior, simultaneous, or after transplant), reason for nephrectomy, approach to nephrectomy (laparoscopic versus open), laterality of nephrectomy (unilateral or bilateral), total operative time and estimated blood loss at the time of nephrectomy and transplant, use of ureteral stents, weight of nephrectomy kidney(s), need for intraoperative or postoperative transfusion, intraoperative complications, need for reoperation, need for readmission, delayed graft function, graft failure, and death. Of note, delayed graft function was defined as the need for dialysis within the first seven days after transplant. The censor date for graft failure and death was January 1, 2013. In addition to these variables, data collected included screening for intracranial aneurysms via CT or MR and form of treatment for those found to have brain aneurysms needing treatment. If available, family history for brain aneurysms was also noted.

In order to assess changes in practice over time, patients were chronologically ordered and divided into two nearly equal groups based on transplant date: (1) early 02/2000–04/2007) and (2) late (04/2007–08/2012). For the purposes of data analysis, for the patients receiving more than one transplant at our institution, each transplant event was counted as a separate case so that outcomes could be analyzed independently. Univariate analysis was performed to look for differences between groups. The independent samples* t*-test was used for continuous variables and Pearson's chi-square or Fisher's exact test was used for categorical variables. Data analysis was performed using IBM Statistical Products and Service Solutions (SPSS, Inc., Chicago Ill) version 21.0 for Windows. The Institutional Review Board of Partners Healthcare approved this study.

## 3. Results

In the 13.5-year study period, data from 133 ADPKD patients who underwent kidney transplantation was collected. Average overall age was 54.1 ± 10.3 years with 37.6% of patients being female. The average number of months of dialysis before transplant was 18.6 ± 21. The majority of transplants were from deceased donors (45.1%), followed by living unrelated donors (34.6%) and living related donors (20.3%). Extended criteria donors were used in nine transplants (6.8%) and donation after cardiac death in 23 transplants (17.3%). All nonheart beating donors were considered controlled. Of the 133 ADPKD patients transplanted, six received a second kidney transplant. Three of the six had their first transplant done at an outside institution. Overall, the average number of days in the hospital after transplant was 5.6 ± 3.5 days.

Indications for pretransplant, simultaneous, and posttransplant nephrectomy are listed in Table 1. Some patients had more than one indication for nephrectomy. Overall, 92 patients (69.2%) received a nephrectomy, some patients receiving more than one. With regard to timing, eight patients (6%) underwent pretransplant native nephrectomy with 7 of these (87.5%) being unilateral. Simultaneous nephrectomy was performed in 74 patients (55.6%) with 67 of these (90.5%) being unilateral. All pretransplant and simultaneous nephrectomies were performed using an open technique. Lastly, 25 patients (18.8%) underwent posttransplant native nephrectomy with 14 of these (56%) being unilateral and 17 being laparoscopic (68%). Combined approaches were done in four patients (3%) who underwent both a pretransplant and simultaneous native nephrectomy and 11 patients (8.3%) who underwent both a simultaneous and posttransplant nephrectomy.

With regard to pretransplant cerebral aneurysm screening, of the 133 patients analyzed in this study, 105 (78.9%) received radiologic imaging (CT or MR) to screen for brain aneurysms. Of those screened, 12 patients (12.4%) were found to have brain aneurysms with seven undergoing surgical treatment with clipping. Data regarding the size of intracranial aneurysms was available for 10 of the 12 cases, with the average size being 3.75 ± 1.41 mm in those undergoing intervention. The location of intracranial aneurysms was diverse and included the anterior communicating artery, middle cerebral artery, posterior cerebral artery, paraclinoid internal carotid artery, and basilar artery. Six patients were found to have a positive family history for brain aneurysm, but none of these patients were found to have brain aneurysm after undergoing radiological screening.

Results of a univariate analysis comparing the first 66 patients in this series (the early group spanning from 02/2000 to 04/2007) to the subsequent 67 patients (the late group spanning from 04/2007 to 08/2012) are present in Tables 2 and 3. There was no significant difference in the donor population utilized between the two eras (Table 2). Of note, significantly more patients were screened for brain aneurysms in the late group (91% versus 66.7%, *P* = 0.001) and although a higher percentage were found to have brain aneurysms in the late group (14.8% versus 6.8%, *P* = 0.207), the difference was not statistically significant. With regard to operative technique, ureteral stents were significantly more common in the late group (64.2% versus 21.2%). Other notable findings include significantly less simultaneous nephrectomies in the late group (22.4% versus 89.4%, *P* < 0.001) and a marginally significant higher proportion of posttransplant nephrectomies (25.4% versus 12.1%, *P* = 0.050). Of the posttransplant nephrectomies, there were significantly less unilateral (35.3% versus 100%, *P* = 0.003) and open (5.9% versus 87.5%, *P* < 0.001) in the late group. The total operative time at transplant was lower in the late group (212.9 minutes ± 59.7 versus 265.0 minutes ± 62.3, *P* < 0.001). Lastly, there was no significant difference between the early and late group with regard to delayed graft function (12.1% versus 16.4%, *P* = 0.479) and mortality (8.2% versus 15%, *P* = 0.242); however, there was a trend towards improved one-year graft survival in the late group (93.1% versus 100%, *P* = 0.05).

Intraoperative complications occurred in 6 patients (4.5%). After transplant, 11 patients (8.3%) required reoperation. Overall, 24 (18%) of patients required readmission within 30 days of discharge. Reasons for readmission are demonstrated in Table 4. With regard to graft and patient outcomes, 19 patients (14.3%) had delayed graft function. The overall mean follow-up was 79.2 ± 108.5 months. Graft failure occurred in 19 patients (14.2%) and the average time to known graft failure was 46.9 ± 41.7 months. Reasons for failure are shown in Table 4. Death occurred in 14 patients (10.5%) and the average time to death was 56.0 ± 44 months. Reasons for death are shown in Table 4.

## 4. Discussion

Of the monogenic disorders, autosomal dominant polycystic kidney disease is the most prevalent [1]. As ADPKD may result in end-stage renal failure, transplantation is often necessary for treatment of this disease. In the current series, 133 patients underwent transplantation for ADPKD at a single center over 13.5 years, with 92 (69.2%) of these patients receiving nephrectomy for indications varying depending upon time of nephrectomy. Over time, simultaneous nephrectomy became less common and posttransplant laparoscopic nephrectomy became the more frequent procedure of choice. With regard to preoperative workup, there was an increasing frequency of screening for intracranial aneurysms in ADPKD recipients. Intraoperative complications remained infrequent; however, the need for readmissions persisted, likely related to the complexity of managing the posttransplant patient. The operative duration shortened in the late time period, likely related to a decreased use of unilateral concomitant native nephrectomy, and there was a trend towards improved one-year graft survival more recently.

Advances in conservative management of ADPKD symptoms with the use of pain medication, antibiotics, transfusion, and antihypertensives [13] as well as minimally invasive methods such as laparoscopic cyst decortication [19] for symptomatic relief have impacted the indications for nephrectomy in this disease [13]. Symptoms such as pain, fullness, early satiety, cystic hemorrhage, hypertension, nephrolithiasis, and urinary tract infections serve as relative indications for nephrectomy. In a single center retrospective review of native nephrectomy in 157 kidney transplant patients with ADPKD, Patel et al. noted that, overall, the most common indication for the 20% of patients undergoing native nephrectomy was urinary tract infection (45%), pain (39%), tumor suspicion (10%), hematuria (3%), and space (3%) [20]. The frequency of these indications was quite different than those identified in the current study in which size was the most common indication for patients undergoing pretransplant and simultaneous nephrectomy, whereas subjective symptoms such as early satiety and chronic pain were the most common indication for those undergoing posttransplant nephrectomy. Furthermore, it should be mentioned that in addition to size considerations, at our institution it was common practice during the early period for surgeons to do simultaneous unilateral nephrectomy in order to then subsequently perform an ureteroureterostomy anastomosis for the transplanted kidney. As surgeons began to perform ureterocystostomy more commonly in the later period, the overall proportion of simultaneous native nephrectomy became less, likely in part to this change in practice.

In addition to changes in the timing and approach to nephrectomy, differences in the use of preoperative screening were noted between the early and late groups. Specifically, in the early group 66.7% of recipients underwent screening compared to 91% in the late groups (*P* < 0.001). There was no significant difference, however, noted in the number found to have brain aneurysms or the number treated for these aneurysms. There are currently no societal guidelines or an established standard of practice to screen for cerebral aneurysms in patients with ADPKD. ADPKD patients with history of subarachnoid hemorrhage, two or more relatives with intracranial aneurysms or rupture, those in high-risk occupations, undergoing major elective surgery, or having a “warning headache” or severe anxiety regarding the issue are suggested to be screened [18]. Specific information regarding screening prior to kidney transplantation is lacking. In a review of presymptomatic screening with magnetic resonance angiography in ADPKD patients, Irazabal et al. found that most unruptured intracranial aneurysms have the growth and rupture risks that are not higher than unruptured intracranial aneurysms found in the general population thus suggesting selective screening [21]. Similarly, a report by Gibbs et al. designed to study the risk of growth and rupture of intracranial aneurysms detected suggests that since most aneurysms detected by such screening are small and have a low risk for rupture, presymptomatic screening should not be done in those without a family history of unruptured intracranial aneurysms [22]. Of note, in the current series, all six patients with a family history underwent screening and none were found to have brain aneurysms. Although there remains a low level of aneurysm detection overall, the majority detected required surgical intervention which may justify the current practice of nonselective radiological assessment until improved screening criteria are developed for patients preparing to undergo kidney transplantation.

With regard to outcomes, it should be noted that over time there was a trend towards improved 1-year graft survival of 100%. Unfortunately this study was not designed to identify independent factors responsible for this observation, but it is possible that some of the above noted changes in practice over time may have contributed. Comparisons of outcomes of ADPKD patients treated with transplantation to other series in the literature are limited in the fact that previously published series often provide outcomes of particular cohorts of patients (e.g., those receiving pretransplant nephrectomy or those undergoing simultaneous nephrectomy and transplantation) as opposed to outcomes for overall experiences. Furthermore, institutional practices vary greatly and given that most reports are of single center retrospective experiences, cross comparisons are difficult.

There are several limitations to this study that are inherent to its being a retrospective single center review. Firstly, this study was limited to variables that were available in the electronic medical records of the patients studied. As this study evaluated changes in practice over time, it should be noted that there was generally more electronic medical information available for patients operated on more recently but more longitudinal follow-up available for patients operated on more remotely, thus possibly introducing bias. Secondly, although this is a large series of patients over a significant time span, the number of patients available for specific stringent statistical analysis was limited due to the prevalence of disease. Therefore, the possibility of Type II error should be considered in subcohort analyses of small sample sizes even though appropriate statistical tests were applied. Thirdly, the observations noted in this study about the change in practice patterns over time reflect those of a single institution that is often dependent on the resources and infrastructure of that particular institution and that may not be directly applicable to the management of this disease at large. Relevant to the current analysis is the fact that it was the practice of this institution to routinely perform ipsilateral nephrectomy at the time of transplant to permit creation of an ureteroneocystostomy. This may have contributed to the high fraction of patients having a nephrectomy with simultaneous transplantation. Lastly, it should be noted that division of groups into early and late was done based on splitting the sample in half rather than splitting the time period in half in order to allow for more statistically even comparisons of groups with regard to total number of patients.

## 5. Conclusions

End-stage renal disease caused by autosomal dominant polycystic kidney disease can effectively be managed by renal transplantation. As a component of therapy, native nephrectomy may also be done but there exists a large degree of variability with respect to timing and approach of nephrectomy. As evidenced by this report, even within the same institution, there has been significant change with an increase in the number of posttransplant, bilateral laparoscopic nephrectomies being performed. Although intraoperative complications, need for reoperation, readmission rates, and mortality did not significantly change over time, there was a trend towards improved 1-year graft survival more recently. We also note a significantly higher use of cerebral imaging over time, with the majority that were detected requiring surgical intervention. As mentioned, this may justify the current practice of nonselective radiological screening until improved screening criteria are developed.

## Figures and Tables

**Table 1:** Indications and timing of nephrectomy.

Indication	Timing of nephrectomy		
	Pretransplant nephrectomy (*n* = 8)	Simultaneous nephrectomy (*n* = 74)	Posttransplant nephrectomy (*n* = 25)
Size	4 (50%)	55 (74.3%)	4 (16%)
Bleeding	2 (25%)	5 (6.8%)	4 (16%)
Subjective symptoms (e.g., early satiety and chronic pain)	3 (37.5%)	4 (5.4%)	14 (56%)
Infection	0	0	4 (16%)
Hypertension	0	0	3 (12%)
Suspicion of neoplasia	0	0	1 (4%)
Hematuria	0	1 (1.4%)	2 (8%)
Not clearly specified	4 (50%)	11 (14.9%)	1 (4%)

Note: some patients had more than one indication for nephrectomy.

**Table 2:** Comparison of categorical variables among the early and late experience in patients receiving transplantation for adult polycystic kidney disease.

Categorical variable	Early (*n* = 66)	Late (*n* = 67)	*P* value
Deceased donor	33 (50%)	27 (40.3%)	0.261
Living related donor	14 (21.2%)	13 (19.4%)	0.795
Living unrelated donor	19 (28.8%)	27 (40.3%)	0.163
Donation after cardiac death	12 (18.2%)	11 (16.4%)	0.788
Extended criteria donor∗	4 (6.1%)	5 (7.5%)	1.000
% male	25 (37.9%)	25 (37.3%)	0.946
Previous native nephrectomy∗	4 (6.1%)	4 (6.0%)	1.000
Previous native nephrectomy was unilateral∗	4 (100%)	3 (75%)	1.000
Screened for brain aneurysms	**44 (66.7%)**	**61 (91%)**	**0.001**
Found to have brain aneurysms	3 (6.8%)	9 (14.8%)	0.207
Treated for brain aneurysms∗	3 (100%)	5 (55.6%)	0.491
Simultaneous native nephrectomy	**59 (89.4%)**	**15 (22.4%)**	**<0.001**
Simultaneous native nephrectomy was unilateral∗	53 (89.8%)	14 (93.3%)	1.000
Simultaneous native nephrectomy was open	59 (100%)	15 (100%)	1.000
Intraop transfusion required	6 (9.1%)	13 (19.4%)	0.089
Side of transplant was on the right	**44 (66.7%)**	**61 (92.4%)**	**<0.001**
Ureteral stent	**14 (21.2%)**	**43 (64.2%)**	**<0.001**
Intraoperative complications∗	3 (4.5%)	7 (10.4%)	0.325
Postop transfusion required	23 (34.8%)	20 (29.9%)	0.538
Need for reoperation	5 (7.6%)	6 (9%)	0.773
Readmission within 30 days	13 (19.7%)	12 (17.9%)	0.792
Posttransplant native nephrectomy	**8 (12.1%)**	**17 (25.4%)**	**0.050**
Posttransplant nephrectomy was unilateral∗	**8 (100%)**	**6 (35.3%)**	**0.003**
Posttransplant nephrectomy was open∗	**7 (87.5%)**	**1 (5.9%)**	**<0.001**
DGF dialysis required	8 (12.1%)	11 (16.4%)	0.479
1-year graft survival∗	54 (93.1%)	62 (100%)	0.052
Death	9 (15%)	5 (8.2%)	0.242

*Fisher's exact test used as ≥1 cell has expected count less than 5.

DGF: delayed graft function.

**Table 3:** Comparison of continuous variables among the early and late experience in patients receiving transplantation for adult polycystic kidney disease.

Continuous variable	Early (*n* = 66)	Late (*n* = 67)	*P* value
Age at transplant (years)	53.7 ± 9.2	54.3 ± 11.3	0.716
Months of dialysis prior to transplant	17.3 ± 18.2	29.2 ± 23.5	0.427
Days in hospital after transplant	6.0 ± 4.4	5.1 ± 2.3	0.158
BMI	25.8 ± 5.9	27.7 ± 4.8	0.076
Previous native nephrectomy total op time (min)	192.3 ± 59.1 in 3 cases	178.3 ± 37.9 in 3 cases	0.747
Previous native nephrectomy EBL (milliliters)	250 ± 70.7 in 2 cases	273.3 ± 46.2 in 3 cases	0.677
Total weight of nephrectomy kidney(s) (grams)	**1536.1 ± 903 in 57 cases**	**3385.6 ± 2304.4 in 28 cases**	**<0.001**
Total op time at time of transplant (min)	**265.0 ± 62.3**	**212.9 ± 59.7**	**<0.001**
Intraop transfusion of RBCs (units)	1.25 ± 0.5 in 4 cases	1.86 ± 1.1 in 7 cases	0.320
Intraop transfusion of FFP (units)	2 ± 0 in 2 cases	3.6 ± 1.5 in 5 cases	0.218
Intraop transfusion of platelets (units)	0	1.5 ± 0.7 in 2 cases	N/A
EBL at time of transplant (milliliters)	332.4 ± 220.5 in 48 cases	257.7 ± 311.5 in 66 cases	0.158
Postop transfusion of RBCs (units)	3.5 ± 3.6 in 23 cases	2.3 ± 1.2 in 17 cases	0.191
Postop transfusion of FFP (units)	7.5 ± 6.4 in 2 cases	2.7 ± 1.2 in 6 cases	0.476
Postop transfusion of platelets (units)	7 ± 1.7 in 3 cases	1 in 1 case	N/A
Posttransplant native nephrectomy total op time (min)	167.3 ± 53.9 in 8 cases	191.8 ± 47.1 in 17 cases	0.257
Posttransplant native nephrectomy total EBL (milliliters)	458.3 ± 488.3 in 6 cases	232.3 ± 311.5 in 15 cases	0.217

BMI: body mass index; EBL: estimated blood loss; RBCs: red blood cells; FFP: fresh frozen plasma.

**Table 4:** Reasons for readmission within 30 days of discharge, graft failure, and death in transplant recipients.

Reasons for readmission (*n* = 24)	Reasons for graft failure (*n* = 19*)	Reasons for death (*n* = 14)
Elevated serum creatinine and/or concern for acute rejection (13, 54.2%)	Acute rejection (3)	Infection (4, 28.6%)
Volume overload (2, 8.3%)	Chronic rejection (3)	Malignancy (3, 21.4%)
Nausea (2, 8.3%)	Calcineurin toxicity (3)	Cardiovascular (1, 7.1%)
Chylocele (1, 4.2%)	Infection (3)	Dementia (1, 7.1%)
Diarrhea (1, 4.2%)	Cardiovascular (2)	Unknown (5, 35.7%)
Peritoneal dialysis catheter removal (1, 4.2%)	Hyperacute rejection (1)	
Perforated duodenal ulcer (1, 4.2%)	Thrombotic microangiopathy (1)	
Bladder stone (1, 4.2%)	Technical (1)	
Wound infection (1, 4.2%)	Malignancy (1)	
Hypophosphatemia (1, 4.2%)	Unknown (3)	

*Frequencies not calculated due to some patients having multiple reasons.
